# The “Heart-and-Brain Interaction” in Newborns with Complex Congenital Heart Disease

**DOI:** 10.1093/ejcts/ezaf367

**Published:** 2025-10-24

**Authors:** Lisa Sogerer-Herold, Charlotte Foltin, David Kronthaler, Daniel Cromb, Milka Pringsheim, Eva Hendrich, Julia Lemmer, Annette Wacker-Gussmann, Christian Meierhofer, Ulrike Held, Serena Counsell, Peter Ewert, Bettina Reich

**Affiliations:** Clinic of Congenital Heart Disease and Pediatric Cardiology, German Heart Center, TUM University Hospital, Technical University of Munich, Munich, 80636, Germany; Clinic of Congenital Heart Disease and Pediatric Cardiology, German Heart Center, TUM University Hospital, Technical University of Munich, Munich, 80636, Germany; Epidemiology, Biostatistics and Prevention Institute, University of Zurich, Zurich, 8001, Switzerland; Centre for the Developing Brain, School of Biomedical Engineering and Imaging Sciences, King’s College London, London, WC2R2LS, United Kingdom; Clinic of Congenital Heart Disease and Pediatric Cardiology, German Heart Center, TUM University Hospital, Technical University of Munich, Munich, 80636, Germany; Institute of Radiology, German Heart Center, TUM University Hospital, Technical University of Munich, Munich, 80636, Germany; Clinic of Congenital Heart Disease and Pediatric Cardiology, German Heart Center, TUM University Hospital, Technical University of Munich, Munich, 80636, Germany; Clinic of Congenital Heart Disease and Pediatric Cardiology, German Heart Center, TUM University Hospital, Technical University of Munich, Munich, 80636, Germany; Clinic of Congenital Heart Disease and Pediatric Cardiology, German Heart Center, TUM University Hospital, Technical University of Munich, Munich, 80636, Germany; Epidemiology, Biostatistics and Prevention Institute, University of Zurich, Zurich, 8001, Switzerland; Centre for the Developing Brain, School of Biomedical Engineering and Imaging Sciences, King’s College London, London, WC2R2LS, United Kingdom; Clinic of Congenital Heart Disease and Pediatric Cardiology, German Heart Center, TUM University Hospital, Technical University of Munich, Munich, 80636, Germany; Clinic of Congenital Heart Disease and Pediatric Cardiology, German Heart Center, TUM University Hospital, Technical University of Munich, Munich, 80636, Germany

**Keywords:** brain volumetry, congenital heart disease, MRI

## Abstract

**Objectives:**

Neonates with complex congenital heart disease (CHD), in particular newborns with hypoplastic left heart syndrome (HLHS) or transposition of the great arteries (TGA), may show brain pathologies and altered brain growth after birth. Our prospective study investigates brain volumes and immature brain structures in these patient groups compared to healthy controls.

**Methods:**

Neonatal cerebral magnetic resonance imaging (MRI) scans were analysed by semi-automated segmentation (dHCP pipeline) in 51 children: 31 HLHS/hypoplastic left heart complex (HLHC)/UVH (61%) at stage I, in 18 neonates with TGA (35%) and in 2 with aortic arch obstruction and biventricular physiology (4%) at a mean GA 41.2 weeks, and in 209 controls at a mean GA 41.6 at time of the MRI. Newborns born premature were excluded. Brain volume comparisons used mixed models for imaging techniques and linear regression for CHD-control differences.

**Results:**

Cerebral MRI was abnormal in 29 patients (57%), with multiple lesions in some patients: including liquor space enlargements (20%), small grey (20%) and white matter injuries (12%), stroke (8%), subdural haemorrhage (22%), and sinus venous thrombosis (8%). Sixty-nine percent of CHD neonates showed signs of brain immaturity in relation to GA. Intracranial volumes were reduced, while cerebrospinal fluid (CSF) volumes were enlarged compared to controls.

**Conclusions:**

Neonates with complex CHD show reduced cerebral growth, higher risk for brain injuries, and impaired brain maturation, even before first surgery. This might constitute a higher perioperative risk in these patient groups than for normal developed brains. Identification of distinct patterns of brain volume loss might enable risk stratification for subsequent neuro-developmental impairment.

**Clinical Registration Number:**

DRKS00036700.

## Graphical abstract

**Figure:**
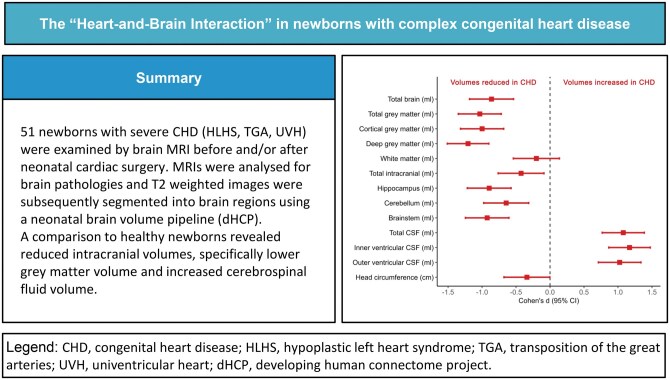

## INTRODUCTION

Children with congenital heart disease (CHD) present with increased risk for neuro-developmental impairments. Brain injury, both, before and after cardiac surgery,^1^ is commonly observed in CHD patients. While these neurological abnormalities have mostly been linked to cerebral vulnerability during the critical neonatal period where open-heart surgery takes place,^2^ increasing evidence suggests that brain impairments in complex CHD fetuses may begin much earlier in utero. Placental pathology^3^ and altered intrauterine haemodynamics^4^^,^^5^ result in an impaired oxygen and nutrition supply to the developing brain.^6^ This affects brain growth during the fetal period,^7^^,^^8^ especially during the last trimester, where human brain growth spurt reaches its peak and brain volume increases 3-4 fold by maturation and gyrification processes.^9^ Hence, delayed brain maturation can result in higher vulnerability of the immature brain cells after birth.^10^ Postnatally, additional factors of early interventions contribute to this process including neonatal intensive care treatments and invasive procedures such as heart catheter interventions and cardiopulmonary bypass surgery.^11^ This can be accompanied by complications, such as disturbed cardiac haemodynamic, prolonged mechanical ventilation, sedation, pain management, treatment of infection, and support by extracorporeal membrane oxygenation (ECMO).^12^

Previous studies have described impaired brain growth and altered brain development in fetuses,^7^^,^^13^ neonates,^10^ and at 2 years of age^14^ in CHD patients.^15^ There seems to be a correlation between brain volumes and poorer neuro-developmental function in children with CHD during infancy,^16^ at 2 years of age,^17^ during adolescence,^18^ and in adulthood.^19^ In this study, the main focus was to evaluate brain damage, brain immaturity, and brain volumes in the highest risk group of neonates with CHD, which require cardiac surgery within the first weeks of life: hypoplastic left heart syndrome (HLHS) and other complex univentricular CHD or neonates with transposition of the great arteries (D-TGA). Recently developed automated sequencing techniques allow for detailed volumetric quantification of different brain compartments in healthy newborns.^20^ Hence, we applied this automated sequencing tool to high-risk CHD newborns, which received their brain magnetic resonance imaging (MRI) in natural sleep without anaesthesia.

The current study had 3 primary objectives. First, to describe brain MRI abnormalities in CHD neonates. Second, to compare brain volumes obtained using 2 different T2 MRI acquisition techniques within CHD neonates. Third, to compare brain volumes between CHD neonates and healthy controls.

## METHODS

### Patients

Between December 2021 and October 2024, neonates (>36 weeks gestational age) with severe CHD, who underwent corrective or palliative surgery within the first 6 weeks of life, were enrolled in the prospective study at the German Heart Centre. For all infants, clinical characteristics were collected and documented. All patients (*n* = 51, 100%) got at least one MRI during their neonatal hospital stay. Brain volume measurement requires additional high quality T2 sequences in three directions (axial, coronal, sagittal; alternatively, one 3D T2 sequence). During natural sleep, it was not always possible to run the complete brain protocol. In those cases, detection of brain pathologies (bleeding, stroke, white matter injury) had higher priority than volumetric measurements.

Age-matched controls were from the third neonatal dHCP data release.^21^ The control sample included 209 infants >37 weeks of gestation, scanned between 37 and 45 weeks PMA and with no major brain lesions reported on their neuroimaging. Exclusion criteria included: admission to the neonatal intensive care unit, a neuro-developmental outcome score below 70 (>2 SD from test mean), a first degree relative with a diagnosed neuro-developmental condition, or multiple pregnancies (e.g. twins).

The study was approved by the ethics committee (2021-321-S-NP, Ethics commission of the Technical University of Munich, Germany) and written parental consent from all participants to use clinical data for study purposes was obtained.

### MRI protocol and image review

**Figure 1.**
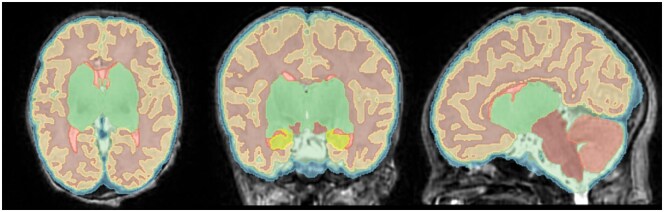
Volumetry Displaying the 9 dHCP Volumes in a Newborn

MRI scans were acquired on 3 T Philips Medical Systems Scanner (Ingenia Elition X) or on 1.5 T Siemens Scanner (Avanto). Infants were swaddled in a warm towel, received noise-protecting ear-plugs, and vital functions were monitored. Most of the scans were performed in natural sleep after feeding. In postoperative scans infants were also scanned in natural sleep after feeding milk and oral chloralhydrate (40 mg/kg), if this was their on-demand medication for weaning of postoperative drugs. MRI-protocols included T1, T2, diffusion (DWI), susceptibility-weighted imaging (SWI), arterial TOF (time-of-flight), and MR-venography. T2 sequences were acquired in axial, sagittal, and coronal orientations. All images were analysed by a neuroradiologist. Any structural abnormalities were recorded and documented. T2 images underwent bias correction using the denoising BTK Toolkit^22^ and subsequently were super-resolution reconstructed using the slice-to-volume reconstruction Toolkit (SVRTK).^23^ The semi-automated dHCP pipeline^20^ was employed to segment the reconstructed 3D MR data. All segmentations were checked visually for quality and minor errors corrected with 3D Slicer^24^ (**Figure 1**). One scan with major segmentation errors was excluded. The analysed intracranial volumes were: cerebrospinal fluid (CSF), cortical gray matter (CGM), white matter (WM), background, ventricles, cerebellum, deep grey matter (GM), brainstem, and hippocampus. We calculated the total brain volume (TBV), total GM (TGM), and CSF adding the respective regional tissue volumes. All volumes were calculated in mL.

### Statistical analysis

Individual characteristics are summarized using descriptive statistics: continuous variables as means and standard deviations (SD) and categorical variables, including missing values, as counts (%). Between-group differences (BGDs) in brain volumes, defined as estimated mean differences between comparison groups, conditional on the model and adjustment variables, were estimated between SVRTK reconstructed and 3D acquired images using mixed effects models including patient-specific intercepts and fixed effects for MRI technique and sex. Fisher’s exact test was used for the evaluation of statistical independence between MRI pathology and type of CHD, surgical intervention, and need for cardiopulmonary bypass in the first week of life, and to test for differences in MRI pathology frequencies between neonates with HLHS and TGA, reporting the odds ratio. Linear regression models were used to estimate BGD in brain volumes between CHD children and healthy controls, adjusting for sex. We report BGDs with 2-sided 95% confidence intervals. Model assumptions were assessed using diagnostic plots: Q-Q plots for normality of residuals and random effects and Tukey-Anscombe plots for homoscedasticity and linearity. All analyses were conducted in R version 4.3.1 in combination with dynamic reporting to guarantee high standards of reproducibility.

## RESULTS

### Descriptive statistics in CHD neonates

**Figure 2.**
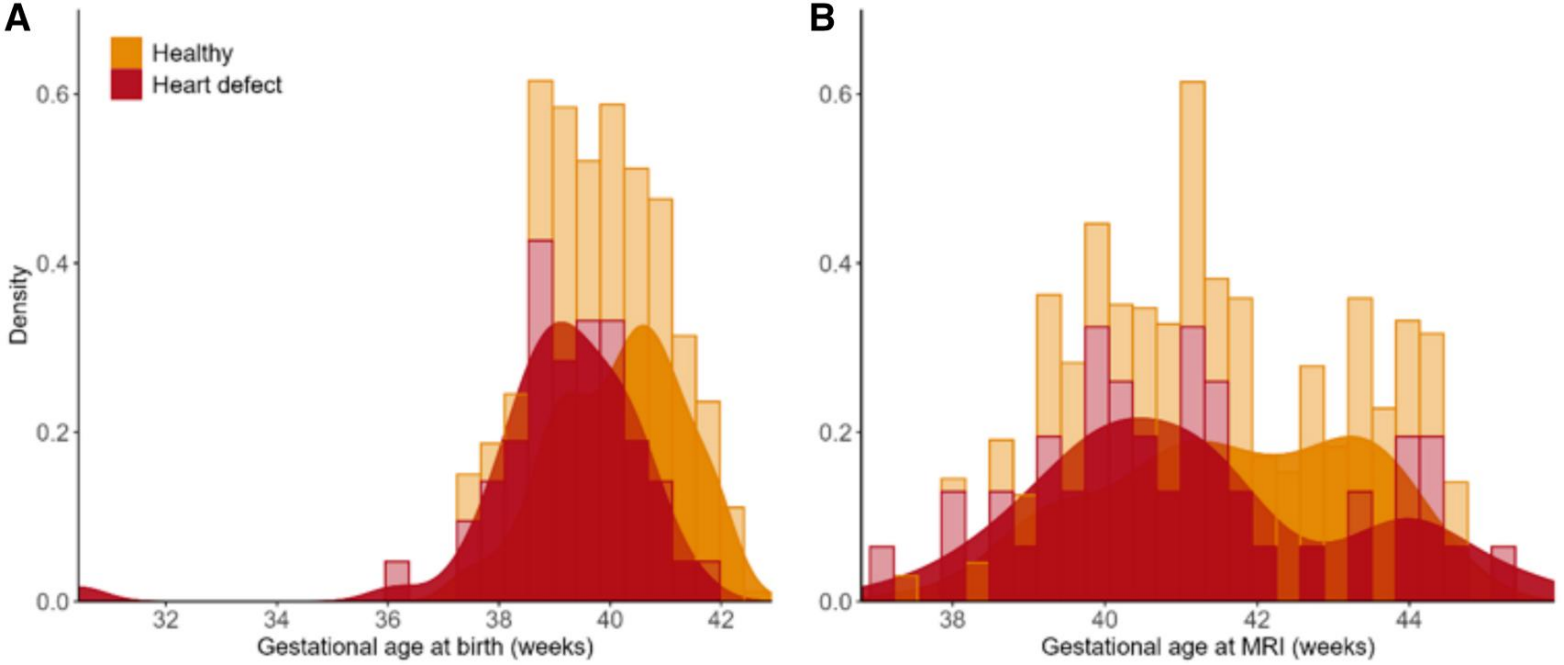
Gestational Ages at Birth (A) und Chronological Gestational Ages of CHD Neonates and Healthy Controls at the Time the MRI Was Performed (B). In cases where pre- and postoperative MRI was done, only the GA at the postoperative timepoint is shown. Abbreviation: MRI: magnetic resonance imaging

Fifty-one CHD neonates were recruited at the German Heart Centre, Munich (42 male, 82%). Two hundred nine age-matched healthy controls were used from the dHCP cohort,^21^ similar to CHD neonates in gestational age at MRI. Individual characteristics are summarized in **Table 1**. At birth, 49% had single-ventricle CHD, 41% biventricular CHD, and 10% borderline ventricle CHD. Ninety-four percent had a cyanotic CHD. The most frequent CHD diagnoses were D-TGA (35%) and HLHS (35%) (**Table 2**). Gestational age at birth are shown in **Figure 2A**. Gestational age at MRI shows 2 peaks in CHD neonates (**Figure 2B**), since MRI was conducted preoperatively or postoperatively.

**Table 1.** Individual Characteristics

Characteristic	*n *= 51	Controls *n* = 209
Sex		
Female	9 (17.6%)	110 (52.6%)
Male	42 (82.4%)	99 (47.4%)
Gestational age at birth	39.1 (1.6)	40.2 (1.2)
Gestational age at MRI	41.2 (2.0)	41.7 (1.7)
Birth weight (g)	3421 (499)	3429 (429)
Birth weight (Z-score)	−0.10 (0.97)	0.20 (1.1)
Scan head circumference (cm)	34.88 (1.56)	35.3 (1.5)
Scan head circumference (Z-score)	−1.11 (1.77)	0.14 (1.25)
Cardiac physiology at birth		
Right-heart dominance	25 (49.0%)	
Left-heart dominance	6 (11.8%)	
Biventricular	20 (39.2%)	
Cyanotic CHD		
Yes	48 (94.1%)	
No	3 (5.9%)	
Antenatal diagnosis		
Yes	33 (64.7%)	
No	18 (35.3%)	
Definitive surgical approach		
Corrective	36 (71.0%)	
Palliative	14 (27.0%)	
Missing	1 (2.0%)	

*n* (%); Mean (SD). Abbreviations: CHD: congenital heart disease; MRI: magnetic resonance imaging

**Table 2.** Cardiac Findings

CHD morphology at birth	Specific type of CHD	*N* = 51
Single ventricle	HLHS	18 (35.3%)
	UVH	10 (19.6%)
Biventricular	D-TGA	18 (35.3%)
	Other (aortic arch hypoplasia, CoA, VSD)	2 (3.9 %)
Borderline	HLHC	3 (5.9%)

*n* (%). Abbreviations: CHD: congenital heart disease; HLHC: hypoplastic left heart complex; HLHS: hypoplastic left heart syndrome; TGA: transposition of the great arteries; UVH: univentricular heart; VSD: ventricular septal defect.

### MRI availability

Brain scans were conventionally analysed in the whole cohort (*n* = 51): In 43 (84%) neonates after heart surgery and in 8 (16%) only before surgery. In 20 children (39%), it was possible to perform 2 MRI scans (pre- and postoperatively) for conventional brain analysis. Due to clinical instability or logistical constraints some pre- or postoperative MRIs could not be conducted.

For specific volumetric measurements of various brain compartments, we were able to analyse this in evaluable quality in 43 children of the cohort of 51 children. In 8 (16%) patients, the 3D T2 sequence was of low quality due to motion artefacts, or the baby woke up too early.

We acquired brain volumetry in 32 neonates (62%) postoperatively and in 11 children (21%) solely before heart surgery. In 13 children (26%), we analysed brain volumetry at two time points, pre- and postoperatively. In those cases, the postoperative scan was used for volumetric analysis in comparison to healthy controls.

### Comparison of MRI acquisition techniques

Comparison of brain volumetry in 3D T2 images vs SVRTK reconstructed T2 images indicated no differences of clinical relevance, with no evidence against differences in analysed brain compartments (**Table S1**).

### Cerebral MRI findings in CHD neonates

Cerebral MRI was pathologic in 57% of the cases that could be mild, moderate or severe pathologies or categorised as single or multiple lesions. Two-third (69%) of the neonatal CHD cohort showed signs of immaturity, such as bilaterally underdeveloped opercular spaces and delayed myelination in relation to gestational age (MRI of the Neonatal Brain, Mary Rutherford, Part 2, Chapter 3, ISBN 0702025348, 2002-2025, and Childs et al^25^) mainly found in patients with hypoplastic aortic arch or d-TGA.

The main findings were liquor space enlargements (20%), small grey (20%) and white matter injuries (12%), stroke (8%), subdural haemorrhage (22%), sinus venous thrombosis (8%), microembolism (12%), and GM injury (16%). Subdural haemorrhages were mainly present on preoperative MR imaging, while intraparenchymal cerebral haemorrhages and sinus venous thrombosis were solely found on postoperative MRI. We found no evidence against statistical independence between MRI pathology and type of CHD (*P* = .97), surgical intervention (*P* = .97), and need for cardiopulmonary bypass in the first week of life (*P* = .62). Postnatally, there was no evidence for differences between HLHS and TGA in terms of frequency of brain damage (odds ratio comparing HLHS to TGA of 0.91, 95% confidence interval (CI) from 0.24 to 3.45; *P* > .99).

**Figure 3.**
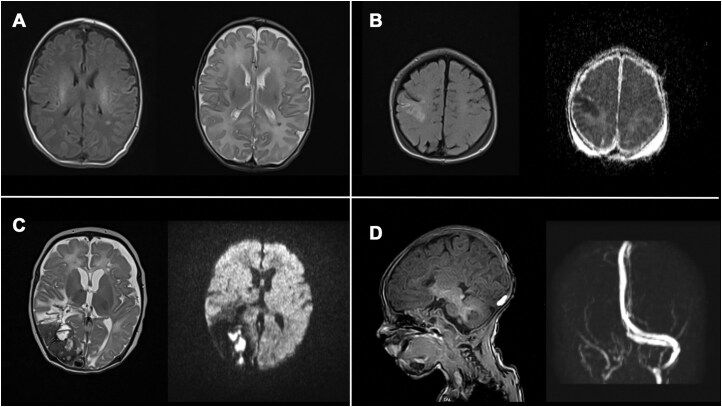
Pathological MRI findings in CHD patients. Preoperative (A, B) and postoperative MRI (C, D). (A) Periventricular WMI on T1 and T2, left parietal, in a newborn with UVH. (B) Subacute infarction in the right hemisphere with involvement of the precentral and postcentral gyrus on T2 and ADC (apparent diffusion coefficient image) in a newborn with D-TGA after balloon atrial septostomy. (C) Extensive colliquation necrosis of the posterior part of the right cerebral hemisphere with a large area of haemorrhages on T2 and ADC in a HLHS patient after hybrid and secondary Norwood I operation. (D) Haemorrhage in the area of the confluens sinuum and underlying sinus vein thrombosis of the right transverse sinus on T1 and MR-venography in a HLHS newborn after hybrid stage I

Most pathological findings were rather small and not very pronounced (**Figure 3A** and **B**), and those infants showed no focal neurological deficit. However, some children (*n* = 3) had severe brain injury postoperatively and multiple lesions with clinical neurological impairments (**Figure 3C**).

### Comparison of CHD neonates and healthy controls

**Figure 4.**
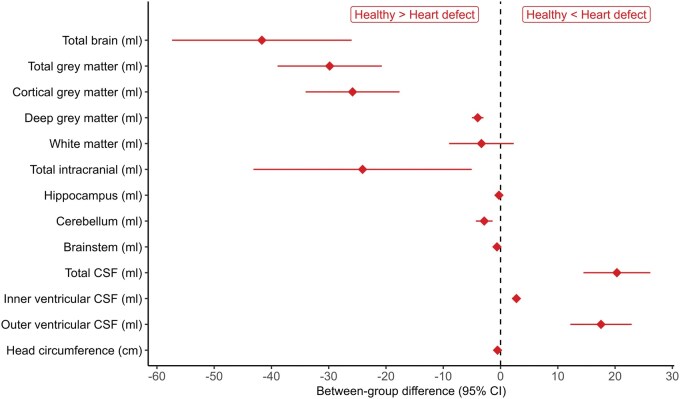
Between-group Differences in Brain Volumes Comparing CHD Neonates to Healthy Controls, Estimated Using Linear Regression Adjusting for Sex. Abbreviation: CSF: cerebrospinal fluid

TBV and TGM were substantially smaller in CHD children, while CSF was considerably enlarged compared to healthy controls, with strong evidence against no differences between groups (**Table 3**, **Figure 4**). Postnatal and in the neonatal period, there was low evidence for white matter volume reduction in CHD patients compared to controls. **Tables S2 and S3** show results of a sensitivity analysis, comparing brain volumes from preoperative and postoperative scans only with healthy controls, with results suggesting robustness across most brain compartments.

**Table 3.** Brain Volume Comparison of CHD Patients and Healthy Controls at a Mean Gestational Age of 41 Weeks

	Controls (*n* = 209)	CHD (*n* = 43)	BGD [SE]	95% CI	*P*	Sex (male vs female) [SE]	95% CI
Total brain volume	376.5 (47.3)	336.6 (40.0)	−41.7 [8.0]	−57.4 to −26.0	<.001	5.8 [6.0]	−6.1 to 17.6
Total grey matter volume	184.7 (27.4)	155.7 (23.3)	−25.8 [4.2]	−34.0 to −17.6	<.001	2.3 [3.2]	−3.9 to 8.5
White matter volume	151.4 (16.2)	149 (18.7)	−3.3 [2.9]	−8.9 to 2.3	.245	3.2 [2.2]	−1.1 to 7.4
Cerebellum volume	26.4 (4.4)	23.6 (3.39)	−2.9 [0.7]	−4.3 to −1.4	<.001	−0.1 [0.6]	−1.2 to 1
Total CSF volume	78.7 (13.6)	99.5 (29.0)	20.3 [3.0]	14.5 to 26.2	<.001	1.6 [2.3]	−2.8 to 6

Mean (SD). Between-group differences (BGDs) with standard errors (SE) and 95% confidence intervals (CIs) in brain volumes were estimated using linear regression adjusting for sex. Abbreviations: CHD: congenital heart disease; CSF: cerebrospinal fluid.

## DISCUSSION

This study provides investigation of cerebral MRI findings and volumetric measurements in high-risk neonates with neonatal cardiopulmonary bypass operation in the first weeks of life. TGA, HLHS, and other complex univentricular heart defects are considered to have the highest risk for brain immaturity, altered brain growth,^8^ and neonatal brain abnormalities.^6^ Often, the postnatal course of these newborns can be aggravated by multiple complications, especially if the heart defect was not known before birth, and due to cardiac interventions (Rashkind manoeuvre, re-opening of the PDA, low cardiac output, lactate acidosis, etc.). All babies received their brain MRI in natural sleep without anaesthesia. We could demonstrate that brain MRI in natural sleep is safe in CHD newborns.

One important finding of the study was that newborns with severe CHD had smaller GM, reduced TBV, and two-third of the neonatal CHD cohort showed signs of immaturity. Moreover, a good half of all patients showed structural cerebral abnormalities on MRI. The most frequent findings were liquor space enlargement, subdural haemorrhage, white matter injury, stroke, and signs of hypoxia/ischaemia. These findings are in line with a retrospective study of the European Association Brain in Congenital Heart Disease Consortium.^1^ Of note, subdural haemorrhage can also be observed in asymptomatic term-born neonates, to a higher extent, if infants underwent instrumental vaginal delivery.^26^ Skotting et al^27^ showed neonatal brain volume differences in a smaller cohort and resumed that in children with CHD the brain impact, which increases the risk of cognitive disabilities. Recently, Peyvandi et al^28^ showed that between 2001 and 2021 the prevalence of postoperative white matter injury (WMI) has declined, whereas preoperative WMI rates remained constant. Improvement of perioperative management may explain these findings and suggest potential modifiable clinical targets to minimise brain injury.

Children with severe CHD have a high risk of cerebral injury, delayed brain development and consequently reduced brain volumes.^16^^,^^29^ MRI scans are the most accurate method for qualitative and quantitative visualisation of structural brain abnormalities. Recent advances in image processing software have led to semi-automated methods with high accuracy and reliability in older children,^30^ healthy newborns,^31^ and newborns with different types of CHD.^17^^,^^32^ In this study, we focused mainly on high-risk CHD (HLHS, UVH, TGA), regarding intrauterine blood flow to the brain. The normal foetal circulation results in preferential streaming of high-oxygenated blood to the brain. This pattern is not present in TGA, where lower oxygenated blood gets to the brain, or in HLHS, where the left ventricle and aorta are hypoplastic and cerebral blood flow is reduced.^33^ During the third trimester foetuses’ brains grow exponentially, and the body shifts energy to the brain to accommodate the neurological growth spurt. As the brain size increases 4-fold, it develops critical structures and connections that will be relied upon for life.^9^ In cases of restricted blood flow to the brain, the foetal autoregulation process, called “brain sparing” effect, redistributes blood flow to the brain,^34^ that can only partially improve the unfavourable situation. In D-TGA the foetal brain receives desaturated blood from the superior and inferior venae cavae. Newborns with D-TGA have small head circumferences with normal birth weight. HLHS newborns are smaller in all dimensions, but head volume is disproportionately decreased.^35^

It is noteworthy that MRI abnormalities, most of them rather small, in our cohort were neither associated with the type of CHD, nor with the type of surgical intervention or the need for neonatal cardiopulmonary bypass.

This is consistent with studies investigating brain development in foetuses and neonates with single-ventricle CHD.^36^ CHD neonates displayed delayed brain development and neurologic abnormalities even before cardiac surgery,^37^ which may partly contribute to reduction of brain volumes, also found in our patients. Neonatal cardiopulmonary bypass itself may result in brain injury due to embolism, cytokine release, and ischaemia, resulting in impaired delivery of energy substrates (oxygen and glucose).^38^

WMI was found in 12% of our patients. While WMI is the characteristic pattern of brain injury in preterm infants, it is increasingly recognised in populations of term newborns with CHD.^39^ Recent findings suggest that the underlying myelination failure observed with WMI is the result of an arrest in the maturation of the oligodentrocyte precursor pool,^40^ leading to an ongoing white matter susceptibility to recurrent insults, such as hypoxia-ischaemia, low cardiac output, or embolic insults. Two major factors predispose the developing brain matter to injury from hypoxia-ischaemia: The presence of arterial end and border zones in the central brain region,^41^ and a propensity for the critically ill neonate to exhibit a pressure-passive circulation related to a disturbance of cerebral autoregulation.^39^ Those insults may further delay brain maturation, aggravate brain injury, and may contribute to our findings of reduced brain volume in our patients.

Significant reduction of white matter will probably only be detectable in a follow-up MRI at 4-6 months of age. These data will be presented in a separate paper. However, two-thirds of our cohort, mainly HLHS and D-TGA neonates, showed signs of brain immaturity already after birth, as described by other groups.^33^

Identification of distinct patterns of brain volume loss might enable risk stratification for subsequent neuro-developmental impairment and may help to improve early intervention for a child development by early intervention programme.

We acknowledge that this work has limitations. Even though all CHD infants had neonatal cardiac surgery, the morphological complexity was differently pronounced, with 2 main groups of HLHS and D-TGA patients. However, we did not find substantial differences in severity nor frequency of brain injury between the groups. There are likely differences in perinatal, perioperative, and surgical management of patients, because infants were born at other clinics, with and without neonatal unit available and transferred to our heart centre at earliest possibility for surgical repair. Nevertheless, this study included a large cohort of infants with complex CHD with comparable preoperative and postoperative imaging and clinical details to enable the investigation of risk factors for brain injury.

During the study period, boys were recruited significantly more often than girls. There was no gender selection of the study group. It is known that there is a significant gender variation in specific CHD subgroups. In particular, there is a significantly greater risk for males to be born with severe CHD and for females with milder CHD subtypes.^42^

CHD and control data were acquired on different scanners in 2 centres, with equal sequences but slightly different parameters. Also, CHD data were acquired on 2 different scanners within the same centre. Even though this might have caused subtle differences in volumetry, we do not assume a significant impact on the differences between groups.

## CONCLUSION

In conclusion, more than half of neonates with high-risk CHD show pathologic MRI findings and signs of brain immaturity for gestational age. Furthermore, intracranial volumes are reduced in the neonatal period compared to healthy controls. Brain MRI in CHD neonates is an excellent tool to visualise focal injuries and structural brain abnormalities and can be performed non-invasively in natural sleep even in neonates with severe CHD. Identification of distinct patterns of brain volume loss might enable risk stratification for subsequent neuro-developmental impairment and may initiate early intervention in the affected individual to improve outcome and quality of life.

## Supplementary Material

ezaf367_Supplementary_Data

## Data Availability

The data underlying this article are available in the article and in its online supplementary material.
