# Supplementary material for: The “Heart-and-Brain Interaction” in Newborns with Complex Congenital Heart Disease
Source: Eur J Cardiothorac Surg. 2025 Oct 24;67(11):ezaf367. doi: 10.1093/ejcts/ezaf367 (PMC12624444; doi:10.1093/ejcts/ezaf367)
Supplement: ezaf367_Supplementary_Data [file ezaf367_supplementary_data.docx]

| **Supplementary Table 1:** Comparison of brain volumes between SVRTK T2 and 3D T2 measurement techniques. Reported are between-group differences (BGD) (3D T2 - SVRTK T2), estimated using mixed models with patient-specific intercepts adjusting for sex (male – female). |
| --- |

| **Volume** | **BGD [SE]** | **95% CI** | **p** | **Male sex [SE]** | **95% CI** |  | **Random Effects SD** | **Residual SD** |
| --- | --- | --- | --- | --- | --- | --- | --- | --- |
| Total brain (ml) | -0.33 [3.03] | -6.42 to 5.76 | 0.91 | 59.01 [26.04] | 8.47 to 109.49 |  | 38.117 | 8.005 |
| Total grey matter (ml) | -2.25 [2.48] | -7.24 to 2.74 | 0.38 | 31.55 [16.8] | -1.06 to 64.1 |  | 24.446 | 6.554 |
| Cortical grey matter (ml) | -2.2 [2.32] | -6.87 to 2.47 | 0.36 | 28.31 [14.97] | -0.75 to 57.32 |  | 21.751 | 6.138 |
| Deep grey matter (ml) | -0.05 [0.22] | -0.49 to 0.39 | 0.82 | 3.25 [2.05] | -0.73 to 7.22 |  | 3.006 | 0.582 |
| White matter (ml) | 2.02 [1.62] | -1.25 to 5.28 | 0.23 | 25.3 [9.08] | 7.67 to 42.88 |  | 13.118 | 4.289 |
| Total intracranial (ml) | -0.83 [3.08] | -7.04 to 5.37 | 0.79 | 72.54 [29.28] | 15.73 to 129.31 |  | 42.925 | 8.154 |
| Hippocampus (ml) | -0.15 [0.04] | -0.23 to -0.07 | 0.002 | 0.2 [0.23] | -0.24 to 0.64 |  | 0.325 | 0.109 |
| Cerebellum (ml) | 0.15 [0.41] | -0.67 to 0.98 | 0.71 | 1.52 [2.44] | -3.21 to 6.24 |  | 3.528 | 1.081 |
| Brainstem (ml) | -0.17 [0.08] | -0.35 to 0 | 0.056 | 0.49 [0.34] | -0.17 to 1.16 |  | 0.482 | 0.225 |
| Total CSF (ml) | -0.4 [1.77] | -3.97 to 3.17 | 0.82 | 15.66 [16.88] | -17.07 to 48.4 |  | 24.741 | 4.693 |
| Inner ventricular CSF (ml) | 0.03 [0.19] | -0.36 to 0.42 | 0.88 | 2.02 [2.3] | -2.44 to 6.47 |  | 3.377 | 0.508 |
| Outer ventricular CSF (ml) | -0.43 [1.62] | -3.7 to 2.84 | 0.79 | 13.64 [14.83] | -15.11 to 42.4 |  | 21.722 | 4.288 |

**Supplementary Table 2:** Comparison of pre-operative brain volumes from CHD patients (N = 24) to healthy controls (N = 209). Between-group differences (BGD, CHD - control) with standard errors (SE) and 95% confidence intervals (CI) were estimated using linear regression adjusting for sex (male – female).

|  | **BGD [SE]** | **95% CI** | **p** | **Male sex [SE]** | **95% CI** |  |
| --- | --- | --- | --- | --- | --- | --- |
| Total brain (ml) | -49.9 [10.26] | -70.12 to -29.68 | < 0.001 | 5.88 [6.24] | -6.41 to 18.17 |  |
| Total grey matter (ml) | -37.21 [5.99] | -49.01 to -25.42 | < 0.001 | 2.16 [3.64] | -5.01 to 9.33 |  |
| Cortical grey matter (ml) | -33.29 [5.39] | -43.91 to -22.67 | < 0.001 | 1.81 [3.28] | -4.64 to 8.27 |  |
| Deep grey matter (ml) | -3.92 [0.65] | -5.21 to -2.64 | < 0.001 | 0.34 [0.4] | -0.44 to 1.13 |  |
| White matter (ml) | -2.72 [3.55] | -9.72 to 4.29 | 0.45 | 3.87 [2.16] | -0.39 to 8.12 |  |
| Total intracranial (ml) | -44.76 [12.25] | -68.9 to -20.62 | < 0.001 | 6.62 [7.45] | -8.05 to 21.3 |  |
| Hippocampus (ml) | -0.23 [0.06] | -0.35 to -0.1 | < 0.001 | 0.08 [0.04] | 0.01 to 0.16 |  |
| Cerebellum (ml) | -4.19 [0.96] | -6.08 to -2.3 | < 0.001 | -0.24 [0.58] | -1.39 to 0.91 |  |
| Brainstem (ml) | -0.48 [0.14] | -0.75 to -0.2 | < 0.001 | 0.04 [0.08] | -0.13 to 0.2 |  |
| Total CSF (ml) | 6.13 [3.3] | -0.36 to 12.63 | 0.06 | 0.65 [2] | -3.3 to 4.6 |  |
| Inner ventricular CSF (ml) | 1.36 [0.38] | 0.6 to 2.11 | < 0.001 | -0.07 [0.23] | -0.52 to 0.39 |  |
| Outer ventricular CSF (ml) | 4.78 [3.05] | -1.23 to 10.78 | 0.12 | 0.71 [1.85] | -2.94 to 4.36 |  |
| Head circumference (cm) | -1.14 [0.34] | -1.8 to -0.48 | < 0.001 | 0.35 [0.2] | -0.05 to 0.75 |  |

**Supplementary Table 3:** Comparison of post-operative brain volumes from CHD patients (N = 32) to healthy controls (N = 209). Between-group differences (BGD, CHD - control) with standard errors (SE) and 95% confidence intervals (CI) were estimated using linear regression adjusting for sex (male -female).

|  | **BGD [SE]** | **95% CI** | **p** | **Male sex [SE]** | **95% CI** |  |
| --- | --- | --- | --- | --- | --- | --- |
| Total brain (ml) | -43.27 [9.09] | -61.18 to -25.36 | <0.001 | 6.22 [6.18] | -5.95 to 18.39 |  |
| Total grey matter (ml) | -30.89 [5.27] | -41.27 to -20.51 | <0.001 | 2.53 [3.58] | -4.52 to 9.58 |  |
| Cortical grey matter (ml) | -26.68 [4.75] | -36.04 to -17.32 | <0.001 | 2.3 [3.23] | -4.06 to 8.66 |  |
| Deep grey matter (ml) | -4.21 [0.58] | -5.35 to -3.07 | <0.001 | 0.23 [0.39] | -0.54 to 1.01 |  |
| White matter (ml) | -3.96 [3.26] | -10.37 to 2.46 | 0.23 | 3.65 [2.21] | -0.7 to 8.01 |  |
| Total intracranial (ml) | -20.64 [10.99] | -42.29 to 1.02 | 0.06 | 7.45 [7.47] | -7.26 to 22.16 |  |
| Hippocampus (ml) | -0.3 [0.06] | -0.41 to -0.19 | <0.001 | 0.07 [0.04] | -0.01 to 0.14 |  |
| Cerebellum (ml) | -2.67 [0.85] | -4.34 to -1 | <0.001 | -0.08 [0.58] | -1.21 to 1.06 |  |
| Brainstem (ml) | -0.68 [0.13] | -0.93 to -0.44 | <0.001 | 0.02 [0.09] | -0.15 to 0.19 |  |
| Total CSF (ml) | 25.67 [3.18] | 19.41 to 31.93 | <0.001 | 1.37 [2.16] | -2.88 to 5.63 |  |
| Inner ventricular CSF (ml) | 3.09 [0.39] | 2.32 to 3.87 | <0.001 | 0.13 [0.27] | -0.39 to 0.66 |  |
| Outer ventricular CSF (ml) | 22.57 [2.92] | 16.82 to 28.33 | <0.001 | 1.24 [1.98] | -2.67 to 5.15 |  |
| Head circumference (cm) | -0.46 [0.3] | -1.06 to 0.14 | 0.13 | 0.32 [0.21] | -0.09 to 0.73 |  |
